# A Bayesian active learning strategy for sequential experimental design in systems biology

**DOI:** 10.1186/s12918-014-0102-6

**Published:** 2014-09-26

**Authors:** Edouard Pauwels, Christian Lajaunie, Jean-Philippe Vert

**Affiliations:** CNRS, LAAS, 7 Avenue du Colonel Roche, Toulouse, F-31400 France; Université de Toulouse LAAS, Toulouse, F-31400 France; MINES ParisTech, PSL-Research University, CBIO-Centre for Computational Biology, 35 rue Saint-Honoré, Fontainebleau, 77300 France; Institut Curie, 26 rue d’Ulm, F-75248, Paris, France; INSERM U900, Paris, F-75248 France

**Keywords:** Systems biology, Kinetic parameter estimation, Active learning, Bayesian experimental design

## Abstract

**Background:**

Dynamical models used in systems biology involve unknown kinetic parameters. Setting these parameters is a bottleneck in many modeling projects. This motivates the estimation of these parameters from empirical data. However, this estimation problem has its own difficulties, the most important one being strong ill-conditionedness. In this context, optimizing experiments to be conducted in order to better estimate a system’s parameters provides a promising direction to alleviate the difficulty of the task.

**Results:**

Borrowing ideas from Bayesian experimental design and active learning, we propose a new strategy for optimal experimental design in the context of kinetic parameter estimation in systems biology. We describe algorithmic choices that allow to implement this method in a computationally tractable way and make it fully automatic. Based on simulation, we show that it outperforms alternative baseline strategies, and demonstrate the benefit to consider multiple posterior modes of the likelihood landscape, as opposed to traditional schemes based on local and Gaussian approximations.

**Conclusion:**

This analysis demonstrates that our new, fully automatic Bayesian optimal experimental design strategy has the potential to support the design of experiments for kinetic parameter estimation in systems biology.

**Electronic supplementary material:**

The online version of this article (doi:10.1186/s12918-014-0102-6) contains supplementary material, which is available to authorized users.

## Background

Systems biology emerged a decade ago as the study of biological systems where interactions between relatively simple biological species generate overall complex phenomena [1]. Quantitative mathematical models, coupled with experimental work, now play a central role to analyze, simulate and predict the behavior of biological systems. For example, ordinary differential equation- (ODE) based models, which are the focus of this work, have proved very useful to model numerous regulatory, signaling and metabolic pathways [2-4], including for example the cell cycle in budding yeast [5], the regulatory module of nuclear factor *κ*B (NF- *κ*B) signaling pathway [6,7], the MAP kinase signaling pathways [8] or the caspase function in apoptosis [9].

Such dynamical models involve unknown parameters, such as kinetic parameters, that one must guess from prior knowledge or estimate from experimental data in order to analyze and simulate the model. Setting these parameters is often challenging, and constitutes a bottleneck in many modeling project [3,10]. On the one hand, fixing parameters from estimates obtained *in vitro* with purified proteins may not adequately reflect the true activity in the cell, and is usually only feasible for a handful of parameters. On the other hand, optimizing parameters to reflect experimental data on how some observables behave under various experimental conditions is also challenging, since some parameters may not be identifiable, or may only be estimated with a large errors, due to the frequent lack of systematic quantitative measurements covering all variables involved in the system; many authors found, for example, that finding parameters to fit experimental observations in nonlinear models is a very ill-conditioned and multimodal problem, a phenomenon sometimes referred to as *sloppiness* [11-17], a concept closely related to that of *identifiability* in system identification theory [18,19], see also [20] for a recent review. When the system has more than a few unknown parameters, computational issues also arise to efficiently sample the space of parameters [21,22], which has been found to be very rugged and sometimes misleading in the sense that many sets of parameters that have a good fit to experimental data are meaningless from a biological point of view [23].

Optimizing the experiments to be conducted in order to alleviate non-identifiabilities and better estimate a system’s parameters therefore provides a promising direction to alleviate the difficulty of the task, and has already been the subject of much research in systems biology [20,24]. Some authors have proposed strategies involving random sampling of parameters near the optimal one, or at least coherent with available experimental observations, and systematic simulations of the model with these parameters in order to identify experiments that would best reduce the uncertainty about the parameters [25-27]. A popular way to formalize and implement this idea is to follow the theory of Bayesian optimal experimental design (OED) [28,29]. In this framework, approximating the model by a linear model (and the posterior distribution by a normal distribution) leads to the well-known A-optimal [30,31] or D-optimal [32-36] experimental designs, which optimize a property of the Fisher information matrix (FIM) at the maximum likelihood estimator. FIM-based methods have the advantage to be simple and computationally efficient, but the drawback is that the assumption that the posterior probability is well approximated by a unimodal, normal distribution is usually too strong. To overcome this difficulty at the expense of computational burden, other methods involving a sampling of the posterior distribution by Monte-Carlo Markov chain (MCMC) techniques have also been proposed [37,38]. When the goal of the modeling approach is not to estimate the parameters *per se*, but to understand and simulate the system, other authors have also considered the problem of experimental design to improve the predictions made by the model [39-41], or to discriminate between different candidate models [42-45].

In this work we propose a new general strategy for Bayesian OED, and study its relevance for kinetic parameter estimation in the context of systems biology. As opposed to classical Bayesian OED strategies which select the experiment that most reduces the uncertainty in parameter estimation, itself quantified by the variance or the entropy of the posterior parameter distribution, we formulate the problem in a decision-theoretic framework where we wish to minimize an error function quantifying how far the estimated parameters are from the true ones. For example, if we focus on the squared error between the estimated and true parameters, our methods attempts to minimize not only the variance of the estimates, as in standard A-optimal designs [30,31], but also a term related to the bias of the estimate. This idea is similar to an approach that was proposed for active learning [46], where instead of just reducing the size of the version space (i.e., the amount of models coherent with observed data) the authors propose to directly optimize a loss function relevant for the task at hand. Since the true parameter needed to define the error function is unknown, we follow an approach similar to [46] and average the error function according to the current prior on the parameters. This results in a unique, well-defined criterion that can be evaluated and used to select an optimal experiment.

In the rest of this paper, we provide a rigorous derivation of this criterion, and discuss different computational strategies to evaluate it efficiently. The criterion involves an average over the parameter space according to a prior distribution, for wich we designed an exploration strategy that proved to be efficient in our experiments. We implemented the criterion in the context of an iterative experimental design problem, where a succession of experiments with different costs is allowed and the goal is to reach the best final parameter estimation given a budget to be spent, a problem that was made popular by the DREAM 6 and DREAM 7 Network Topology and Parameter Inference Challenge [47-49]. We demonstrate the relevance of our new OED strategy on a small simulated network in this context, and illustrate its behavior on the DREAM7 challenge. The method is fully automated, and we provide an R package to reproduce all simulations.

## Methods

### A new criterion for Bayesian OED

In this section we propose a new, general criterion for Bayesian OED. We consider a system whose behavior and observables are controlled by an unknown parameter $\theta ^{*}\in \Theta \subset \mathbb {R}^{p}$ that we wish to estimate. For that purpose, we can design an experiment *e*∈, which in our application will include which observables we observe, when, and under which experimental conditions. The completion of the experiment will lead to an observation *o*, which we model as a random variable generated according to the distribution *o*∼*P*(*o*|*θ*^∗^;*e*). Note that although *θ*^∗^ is unknown, the distribution *P*(*o*|*θ*;*e*) is supposed to be known for any *θ* and *e*, and amenable to simulations; in our case, *P*(*o*|*θ*;*e*) typically involves the dynamical equations of the system if the parameters are known, and the noise model of the observations.

Our goal is to propose a numerical criterion to quantify how “good” the choice of the experiment *e* is for the purpose of evaluating *θ*^∗^. For that purpose, we assume given a loss function *ℓ* such that *ℓ*(*θ*,*θ*^∗^) measures the loss associated to an estimate *θ* when the true value is *θ*^∗^. A typical loss function is the squared Euclidean distance *ℓ*(*θ*,*θ*^∗^)=∥*θ*−*θ*^∗^∥^2^, or the squared Euclidean distance in after a log transform for positive parameters $\ell (\theta,\theta ^{*})=\sum _{i=1}^{p} \log (\theta _{i} / \theta ^{*}_{i})^{2}$. We place ourselves in a Bayesian setting, where instead of a single point estimate the knowledge about *θ*^∗^ at a given stage of the analysis is represented by a probability distribution *π* over *Θ*. The quality of the information it provides can be quantified by the average loss, or risk:

$$E_{\theta \sim \pi(\theta)} \ \ell(\theta,\theta^{*}) = \int \ell(\theta,\theta^{*})\,\pi(\theta)\, d\theta\,. $$

Once we choose an experiment *e* and observe *o*, the knowledge about *θ*^∗^ is updated and encoded in the posterior distribution

(1) $$ P(\theta|o;e)\ =\ \frac{P(o|\theta;e)\,\pi(\theta)}{\int_{\theta^{'}}P(o|\theta^{'};e)\,\pi(\theta^{'})d\theta^{'}}\,,  $$

whose risk is now:

$$\begin{array}{*{20}l} &E_{\theta \sim P(\theta|o;e)} \ \ell(\theta,\theta^{*}) \\ &= \int_{\theta} \ell(\theta,\theta^{*})\,\frac{P(o|\theta;e)\,\pi(\theta)}{\int_{\theta^{'}}P(o|\theta^{'};e)\,\pi(\theta^{'})d\theta^{'}}\ d\theta\,. \end{array} $$

The above expression is for a particular observation *o*. This observation is actually generated according to *P*(*o*|*θ*^∗^;*e*). Accordingly, the average risk of the experiment *e* (if the true parameter is *θ*^∗^) is:

$$E_{o\sim P(o|\theta^{*};\,e)}E_{\theta \sim P(\theta|o;e)} \ \ell(\theta,\theta^{*}) \,. $$

Finally, *θ*^∗^ being unknown, we average the risk by taking account of the current state of knowledge, and thus according to *π*. The expected risk associated to the choice of *e* when the current knowledge about *θ*^∗^ is encoded in the distribution *π* is thus:

(2) $$\begin{array}{*{20}l} & R(e ; \pi) \\ &= E_{\theta^{'}\sim \pi(\theta^{'})} E_{o\sim P(o|\theta^{'};\,e)}E_{\theta \sim P(\theta|o;e)} \ \ell(\theta,\theta^{'})\\ &=\\ &\int_{\theta,\theta^{'}} \ell(\theta,\theta^{'})\,\int_{o}\frac{P(o|\theta;e)\,\pi(\theta)\, P(o|\theta^{'};e)\,\pi(\theta^{'})}{\int_{\theta^{{'}{'}}}P(o|\theta^{{'}{'}};e)\,\pi(\theta^{{'}{'}})d\theta^{{'}{'}}}\, d\theta\, d\theta^{'} \,.  \end{array} $$

The expected risk *R*(*e*;*π*) of a candidate experiment *e* given our current estimate of the parameter distribution *π* is the criterion we propose in order to assess the relevance of performing *e*. In other words, given a current estimate *π*, we propose to select the best experiment to perform as the one that minimizes *R*(*e*;*π*). We describe in the next section more precisely how to use this criterion in the context of sequential experimental design where each experiment has a cost.

Note that the criterion *R*(*e*;*π*) is similar but different from classical Bayesian OED criteria, like the variance criterion used in A-optimal design. Indeed, taking for example the square Euclidean loss as loss function *ℓ*(*θ*,*θ*^∗^)=∥*θ*−*θ*^∗^∥^2^, and denoting by *π*_*e*_ the mean posterior distribution that we expect if we perform experiment *e*, standard A-optimal design tries to minimize the variance of *π*_*e*_, while our criterion focuses on:

$$E_{\theta \sim \pi_{e}} \ell(\theta,\theta^{*}) = \| E_{\theta\sim\pi_{e}}[\theta]- \theta^{*}\|^{2} + \text{Var}(\pi_{e})\,. $$

In other words, our criterion attempts to control both the bias and the variance of the posterior distribution, while standard Bayesian OED strategies only focus on the variance terms. While both criteria coincide with unbiased estimators, there is often no reason to believe that the estimates used are unbiased.

### Sequential experimental design

In sequential experimental design, we sequentially choose an experiment to perform, and observe the resulting outcome. Given the past experiments *e*_1_,…,*e*_*k*_ and corresponding observations *o*_1_,…,*o*_*k*_, we therefore need to choose what is the best next experiment *e*_*k*+1_ to perform, assuming in addition that each possible experiment *e*∈ has an associated cost *C*_*e*_ and we have a limited total budget to spend.

We denote by *π*_*k*_ the distribution on *Θ* representing our knowledge about *θ*^∗^ after the *k*-th experiment and observation, with *π*_0_ representing the prior knowledge we may have about the parameters before the first experiment. According to Bayes’ rule (1), the successive posteriors are related to each other according to:

$$\pi_{i+1}(\theta) = \frac{P(o_{i+1}|\theta;e_{i+1})\,\pi_{i}(\theta)}{\int_{\theta^{'}}P(o_{i+1} |\theta^{'};e_{i+1})\,\pi_{i}(\theta^{'})d\theta^{'}}\,. $$

Although a global optimization problem could be written to optimize the choice of the *k*-th experiment based on possible future observations and total budget constraint, we propose a simple, greedy formulation where at each step we choose the experiment that most decreases the estimation risk per cost unit. If the cost of all experiments were the same, this would simply translate to:

$$e_{k+1} = \arg\min_{e\in{\cal E}} R(e;\pi_{k})\,. $$

To take into account the different costs associated with different experiments, we consider as a baseline the mean risk when the knowledge about *θ*^∗^ is encoded in a distribution *π* over *Θ*:

$$R(\pi) = E_{\theta \sim \pi(\theta)} E_{\theta^{'} \sim \pi(\theta^{'})} \ \ell(\theta,\theta^{'})\,, $$

and choose the experiment that maximally reduces the expected risk per cost unit according to:

(3) $$ e_{k+1} = \arg\max_{e\in{\cal E}} \ \frac{R(\pi_{k}) - R(e;\pi_{k})}{C_{e}}\,.  $$

### Evaluating the risk

The expected risk of an experiment *R*(*e*;*π*) (2) involves a double integral over the parameter space and an integral over the possible observations, a challenging setting for practical evaluation. Since no analytical formula can usually be derived to compute it exactly, we now present a numerical scheme that we found efficient in practice. Since the distribution *π*_*k*_ over the parameter space after the *k*-th experiment can not be manipulated analytically, we resort on sampling to approximate it and estimate the integrals by Monte-Carlo simulations.

Let us suppose that we can generate a sample *θ*_1_,…,*θ*_*N*_ distributed according to *π*. Obtaining such a sample itself requires careful numerical considerations discussed in the next section, but we assume for the moment that it can be obtained and show how we can estimate *R*(*e*;*π*) from it for a given experiment *e*. For that purpose, we write

$$w_{ij}(e)\,=\,\int_{o}\frac{P(o|\theta_{i};e)\, P(o|\theta_{j};e)}{\sum_{k=1}^{N}P(o|\theta_{k};e)}\, do $$

for 0≤*i*,*j*≤*N*, as a discrete estimate of the second integral in equation (2). Since $\{\theta _{i}\}_{i=1}^{N}$ are independantly drawn from *π* the prior terms disappear. Moreover, the denominator is a discretization of the denominator in equation (2), and the likelihood *P* is supposed to be given. We have the standard estimate of (2) by an empirical average:

(4) $$ R^{N}(e;\pi)\,=\,\frac{1}{N^{2}}\sum_{i,j=1}^{N} \ell(\theta_{i},\theta_{j})\, w_{ij}(e)\,.  $$

We see that the quantity *w*_*ij*_(*e*) measures how similar the observation profiles are under the two alternatives *θ*_*i*_ and *θ*_*j*_. A good experiment produces dissimilar profiles and thus low values of *w*_*ij*_(*e*) when *θ*_*i*_ and *θ*_*j*_ are far appart. The resulting risk is thus reduced accordingly.

For each *i* and *j*, the quantity *w*_*ij*_(*e*) can in turn be estimated by Monte-Carlo simulations. For each *θ*_*i*_, a sample of the conditionnal distribution *P*(*o*|*θ*_*i*_;*e*), denoted by ${o_{u}^{i}}$ (*u*=1,⋯,*M*) is generated. The corresponding approximation is:

(5) $$ w_{ij}^{M}(e)\ =\ \frac{1}{M}\sum_{u=1}^{M}\frac{P({o_{u}^{i}}|\theta_{j};e)}{\sum_{k=1}^{N} P({o_{u}^{i}}|\theta_{k};e)}\,,  $$

which can be interpreted as a weighted likelihood of the alternative when the observation is generated according to *θ*_*i*_.

In most settings, generating a sample ${o_{u}^{i}}$ involves running a deterministic model, to be performed once for each *θ*_*i*_, and degrading the output according to a noise model independently for each *u*. In our case, we used the solver proposed in [50] provided in the package [51] to simulate the ODE systems. Thus, a large number *M* can be used if necessary at minimal cost. Based on these samples, the approximated weights $w_{\textit {ij}}^{M}$ can be computed from (5), from which the expected risk of experiment *e* can be derived from (4).

Note that an appealing property of this scheme is that the same sample *θ*_*i*_ can be used to evaluate all experiments. We now need to discuss how to obtain this sample.



### Sampling the parameter space

**Figure 1:**
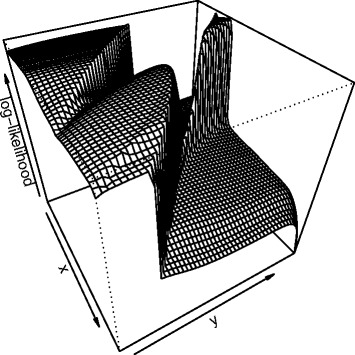
**Log likelihood surface.** Log likelihood surface for parameters living on a restricted area of a two dimensional plane. For clarity, scale is not shown. Areas with low log-likelihood correspond to dynamics that do not fit the data at all, while areas with high log-likelihood fit the data very well. The surface shows multi-modality, plateaus and abrupt jumps which makes it difficult to sample from this density. When parameters do not live on a plane, these curses have even higher effect.

Sampling the parameter space according to *π*_*k*_, the posterior distribution of parameters after the *k*-th experiment, is challenging because the likelihood function can exhibit multi-modality, plateaus and abrupt changes as illustrated in Figure 1. Traditional sampling techniques tend to get stuck in local optima, not accounting for the diversity of high likelihood areas of the parameter space [52]. In order to speed up the convergence of sampling algorithm to high posterior density regions, we implemented a Broyden-Fletcher-Goldfarb-Shanno (BFGS) quasi-Newton optimization algorithm using finite difference approximation for gradient estimation [53] in order to identify several modes of the posterior distribution, and used these local maxima as initial values for a Metropolis Hastings sampler, combining isotropic Gaussian proposal and single parameter modifications [52]. We then use a Gaussian mixture model approximation to estimate a weighting scheme of in order to account for the initialization process when recombining samples from different modes. Annex B, given in the Additional file 1 provides computational details for this procedure.

The method described in Algorithm ?? is independant of the sampling scheme used. However, convergence of posterior samples is essential to ensure a good behaviour of the method. First, it is known that improper (or “flat”) priors may lead to improper posterior distributions when the model contains non identifiabilities. Such issues should be avoided since MCMC based sampling schemes are known not to converge in these cases. Therefore, proper prior distributions are essential in this context and improper priors should not be used in order to avoid improper posteriors. The second important element for posterior samples is numerical convergence of the sampling scheme, usually guaranteed asymptotically. Fine tuning parameters that drive the scheme is necessary to ensure that one is close to convergence in a reasonable amount of time. To check appropriate sampling behaviour, we use a graphical heuristic. We draw ten different samples from the same posterior distribution, using different initialization seeds. For each model parameter, we compare the dispersion within each sample to the total dispersion obtained by concatenating the ten samples. This value should be close to one. Such an heuristic can be used to tune parameters of the sampler, such as sample size or proposal distribution. More details and numerical results are given in Additional file 1: Annex B.

### Enforcing regularity through the prior distribution

The prior distribution *π*_0_ plays a crucial role at early stages of the design, as it can penalize parameters leading to dynamical behaviors that we consider unlikely. In addition to a large variance log normal prior, we considered penalizing parameters leading to non smooth time trajectories. This is done by adding to the prior log density a factor that depends on the maximum variation of time course trajectories as follows. To each parameter value *θ* are associated trajectories, *Y*_*i*,*t*_, which represent concentration values of the *i*-th species at time *t*. In the evaluation of the log prior density at *θ*, we add a term proportional to

$$\max_{i,t} (Y_{i,t+1} - Y_{i,t})^{2}. $$

The advantage of this is twofold. First, it is reasonable to assume that variables we do not observe in a specific design vary smoothly with time. Second, this penalization allows to avoid regions of the parameter space corresponding to very stiff systems, which are poor numerical models of reality, and which simulation are computationally demanding or simply make the solver fail. This penalty term is only used in the local optimization phase not during the Monte Carlo exploration of the posterior. The main reason for adopting such a scheme is numerical stability.

The choice of prior parameters directly affects the posterior disribution, specially when a low amount of data is available. In our experiments, the prior is chosen to be log-normal with large variance. This allows to cover a wide range of potential physical values for each parameter (from 10^−9^ to 10^9^). The weight of the regularity enforcing term has also to be determined. Since the purpose is to avoid regions corresponding to numerically unstable systems, we chose this weight to be relatively small compared to the likelihood term. In practical applications, parameters have to be chosen by considering the physical scale of quantities to be estimated. Indeed, a wrong choice of hyper parameter leads to very biased estimates at the early stages of the design.

## Results and discussion

### In silico network description

**Figure 2:**
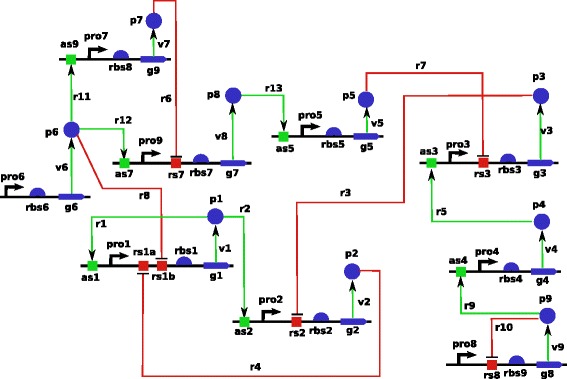
**Gene network for DREAM7 Challenge.** Gene network for DREAM7 Network Topology and Parameter Inference Challenge. Promoting reactions are represented by green arrows and inhibitory reactions are depicted by red arrows.

In order to evaluate the relevance of our new sequential Bayesian OED strategy in the context of systems biology, we test it on an *in silico* network proposed in the DREAM7 Network Topology and Parameter Inference Challenge which we now describe [49]. The network, represented graphically in Figure 2, is composed of 9 genes and its dynamics is governed by ordinary differential equations representing kinetic laws involving 45 parameters. Promoting reactions are represented by green arrows and inhibitory reactions are depicted by red arrows. For each of the 9 genes, both protein and messenger RNA are explicitly modelled and therefore the model contained 18 continuous variables. Promoter strength controls the transcription reaction and ribosomal strength controls the protein synthesis reaction. Decay of messenger RNA and protein concentrations is controlled through degradation rates. A complete description of the underlying differential equations is found in Additional file 2: Annex A. The complete network description and implementations of integrators to simulate its dynamics are available from [49].

Various experiments can be performed on the network producing new time course trajectories in unseen experimental conditions. An experiment consists in choosing an action to perform on the system and deciding which quantity to observe. The possible actions are

- do nothing (wild type);
- delete a gene (remove the corresponding species);
- knock down a gene (increase the messenger RNA degradation rate by ten folds);
- decrease gene ribosomal activity (decrease the parameter value by 10 folds).

These actions are coupled with 38 possible observable quantities

- messenger RNA concentration for all genes, at two possible time resolutions (2 possible choices);
- protein concentration for a single pair of proteins, at a single resolution (resulting in 9∗8/2=36 possible choices).

Purchasing data consists in selecting an action and an observable quantities. In addition, it is possible to estimate the constants (binding affinity and hill coefficient) of one of the 13 reactions in the system. Different experiments and observable quantities have different costs, the objective being to estimate unknown parameters as accurately as possible, given a fixed initial credit budget. The cost of the possible experiments are described in Table S1 in Additional file 2: Annex A.

For simulation purposes, we fix an unknown parameter value *θ*^∗^ to control the dynamics of the systems, and the risk of an estimator is defined in terms of the loss function $\ell (\theta,\theta ^{*}) = \sum _{i=1}^{p} \log \left (\theta _{i} / \theta ^{*}_{i} \right)^{2}$.

The noise model used for data corruption is heteroscedastic Gaussian: given the true signal $y \in \mathbb {R}^{+}$, the corrupted signal has the form *y*+*z*_1_+*z*_2_, where *z*_1_ and *z*_2_ are centered normal variables with standard deviation 0.1 and (0.2×*y*), respectively.

### Performance on a 3-gene subnetwork

In order to assess the performance of our sequential OED strategy in an easily reproducible setting, we first compare it to other strategies on a small network made of 3 genes. We take the same architecture as in Figure 2, only considering proteins 6, 7 and 8. The resulting model has 6 variables (the mRNA and protein concentrations of the three genes) whose behavior is governed by 9 parameters. There are 50 possible experiments to choose from for this sub network: 10 perturbations (wildtype and 3 perturbations for each gene) and 5 observables (mRNA concentrations at two different time resolutions and each protein concentration at a single resolution). We compare three ways to sequentially choose experiments in order to estimate the 9 unknown parameters: (i) our new Bayesian OED strategy, including the multimodal sampling of parameter space, (ii) the criterion proposed in equation (13) in [27] together with our posterior exploration strategy, and (iii) a random experimental design, where each experiment not done yet is chosen with equal probability. The comparison of (i) and (ii) is meant to compare our strategy with a criterion that proved to be efficient in a similar setting. The comparison to (iii) is meant to assess the benefit, if any, of OED for parameter estimation in systems biology. Since all methods involve randomness, we repeat each experiment 10 times with different pseudo-random number generator seeds.

**Figure 3:**
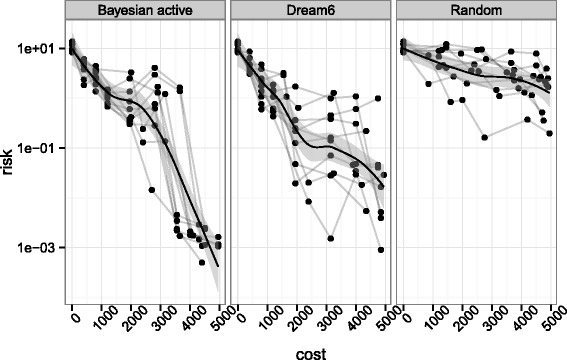
**Comparison of risk evolution between different strategies.** Comparison of risk evolution between different strategies on a subnetwork. The figure shows the true risk at each step of the procedure, *i.e.* the approximate posterior distribution is compared to the true underlying parameter which is unknown during the process. The risk is computed at the center of the posterior sample. The different lines represent 10 repeats of the design procedure given the same initial credit budget and the points represent experiment purchase. The first panel represents our strategy, the second panel implements the criterion of the best performing team on DREAM6 challenge while random design consists in choosing experiments randomly.

The results are presented in Figure 3, where we show, for each of the three methods, the risk of the parameter estimation as a function of budget used. Here the risk is defined as the loss between the true parameter *θ*^∗^ (unknown to the method) and the estimated mean of the posterior distribution. After *k* rounds of experimental design, one has access to *k* experimental datasets which define a posterior distribution *π*_*k*_ from which a sample $\{{\theta _{i}^{k}}\}_{i=1}^{N}$ is drawn. The quantities displayed in Figure 3 are computed as

$$\mathbb{E}_{\theta \sim \pi_{k}(\theta)}\left[ \ell(\theta, \theta^{*}) \right] \simeq \frac{1}{N} \sum_{i=1}^{n} \ell({\theta_{i}^{k}}, \theta^{*}), $$

which would be the true risk that one would have to support. We first observe that the random sampling strategy has the worst risk among the three strategies, suggesting that optimizing the experiments to be made for parameter estimation outperforms a naive random choice of experiments. Second, and more importantly, the comparison between the first and second panel suggests that, given the same parameter space exploration heuristic, our proposed strategy outperforms the criterion given in [27]. It is worth noting that this criterion is part of a strategy that performed best in DREAM6 parameter estimation challenge. Although a large part of their design procedure involved human choice which we did not implement, we reproduced the part of their procedure that could be automatised. A compagnon of Figure 3 is given in Figure S3 in Additional file 1: Annex B where we illustrate based on parameter samples how lacks of identifiability manifest themselves in a Bayesian context and how the design strategy alleviates them in terms of posterior distribution. In summary, this small experiment validates the relevance of our Bayesian OED strategy.

### Results on the full DREAM7 network

**Figure 4:**
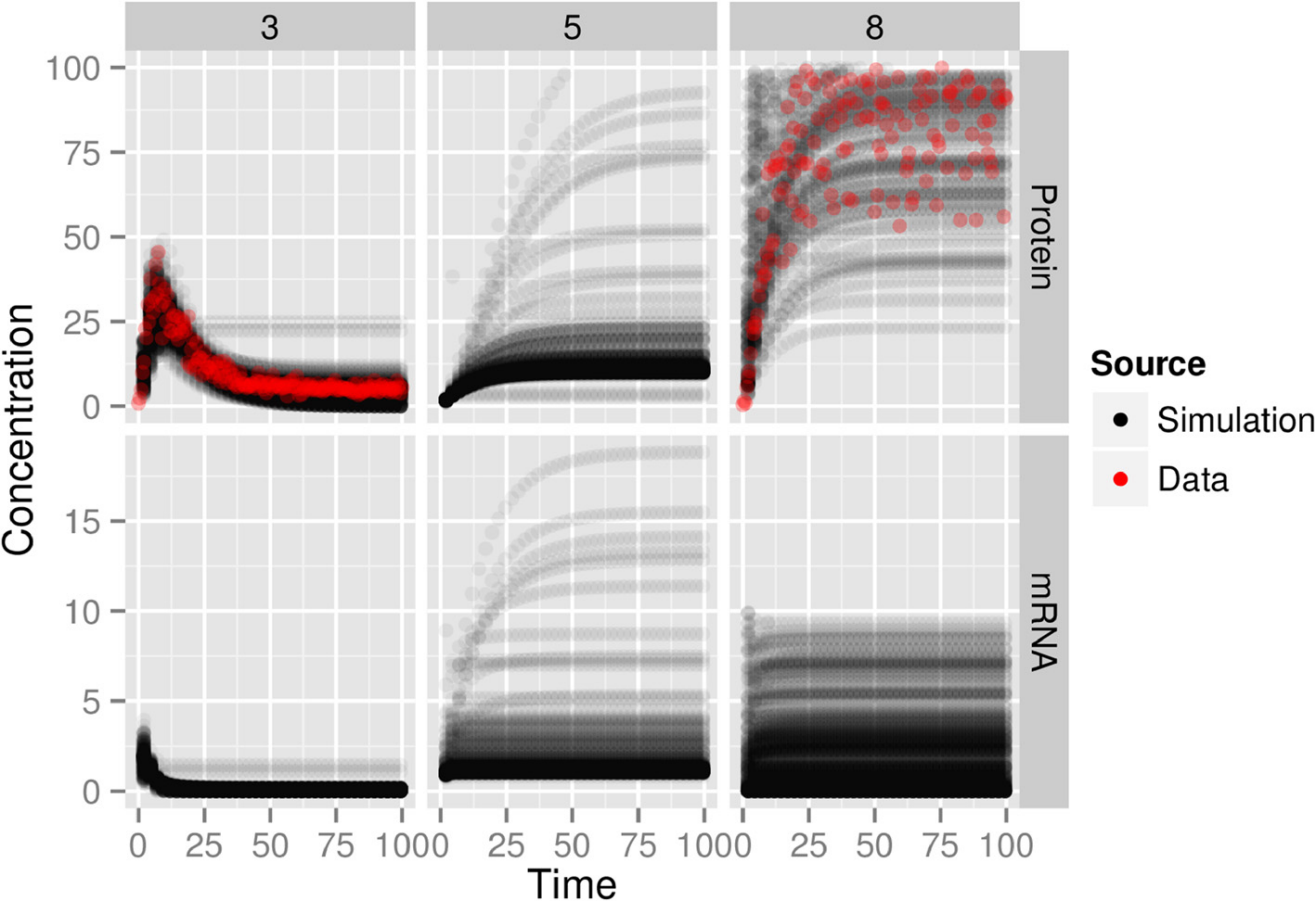
**Trajectories from posterior sample.** Corresponds to Table 1 figures. We plot trajectories from our posterior sample (protein 8 concentration was divided by 2 and we do not plot concentrations higher than 100). The quantities with the highest variability are protein 8 and 3 concentrations. This is consistent with the estimated risks in Table 1. There is quite a bit of uncertainty in protein 5 concentration, however this is related to protein 8 uncertainty as protein 8 is an inhibitor of protein 5. Moreover, mRNA concentration have much lower values and are not as informative as proteins concentrations. Red dots shows the data we purchased for this experiment after seeing these curve and in accordance with results in Table 1.

To illustrate the behavior of our OED strategy in a more realistic context, we then apply it to the full network of Figure 2 following the setup of the DREAM7 challenge. At the beginning of the experiment, we already have at hand low resolution mRNA time courses for the wild type system. The first experiments chosen by the method are wild-type protein concentration time courses for all genes. The detailed list of purchased experiments is found Table S2 in Additional file 2: Annex A. This makes sense since we have enormous uncertainty about proteins time courses, given that we do not know anything about them. Once these protein time series are purchased, the suggestion for the next experiment to carry out is illustrated in Table 1. Interestingly, the perturbations with the lowest risk are related to gene 7 which is on the top of the cascade (see Figure 2). Moreover it seemed obvious from Table 1 that we have to observe protein 8 concentration. Indeed, Figure 4 shows that there is a lot of uncertainty about protein 8 evolution when we remove gene 7.

**Table 1:** **Estimation of the expected risk**

**Risk**	**Cost**	**Experiment**	**Observe proteins**
771	1200	Delete gene 7	3-8
1196	850	Decrease gene 7 RBS activity	3-8
1290	750	Knock down gene 7	3-8
1957	850	Decrease gene 7 RBS activity	3-7
2254	850	Decrease gene 7 RBS activity	7-8
2554	1200	Delete gene 9	3-8
2867	750	Knock down gene 7	8-9
4647	1200	Delete gene 7	8-9
4798	850	Decrease gene 7 RBS activity	8-9
4928	850	Decrease gene 7 RBS activity	5-8

Estimation of the expected risk at a certain stage of the experimentation, ten lowest values. There is consistency in the type of experiment to be conducted (targeting gene 7 which expression impacts on a big part of the network) and the quantities to measure (protein 8 almost all the time and protein 3 quite often). Figure 4 illustrate this point further.

Moreover, our criterion determines that it is better to observe protein 3 than protein 5, which makes sense since the only protein which affects protein 5 evolution is protein 8 (see Figure 2). Therefore uncertainty about protein 5 time course is tightly linked to protein 8 time course, and observing protein 3 brings more information than observing protein 5. This might not be obvious when looking at the graph in Figure 4 and could not have been foreseen by a method that considers uncertainty about each protein independently. At this point, we purchase protein 3 and 8 time courses for gene 7 deletion experiment and highlight in red in Figure 4 the profiles of proteins 3 and 8 obtained from the system.

**Figure 5:**
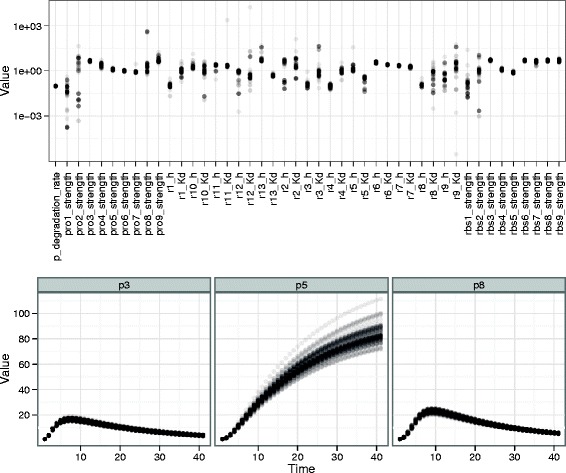
**Comparison of parameter and trajectory variability.** Comparison of parameter variability and time course trajectory variability. This is a sample from the posterior distribution after spending all the credits in the challenge. The top of the figure shows parameter values on log scale, while the bottom shows prediction of protein time courses for an unseen experiment. The range of some parameter values is very wide while all these very different values lead to very similar protein time course predictions.

In addition to parameter estimation, one may be interested in the ability of the model with the inferred parameters to correctly simulate time series under different experimental conditions. Figure 5 represents a sample from the posterior distribution after all credits have been spent (unseen experiment description is given in Table S3 Additional file 2: Annex A). Both parameter values and protein time course for the unseen experiment are presented.

Some parameters, like *p*_*degradation*_*rate* or *pro*3_*srenght*, clearly concentrate around a single value while others, like *pro*1_*strength* or *pro*2_*strength*, have very wide ranges with multiple accumulation points. Despite this variability in parameter values, the protein time course trajectories are very similar. It appears that protein 5 time course is less concentrated than the two others. This is due to the hetroscedasticity of the noise model which was reflected in the likelihood. Indeed, the noise model is Gaussian with standard deviation increasing with the value of the corresponding concentration. Higher concentrations are harder to estimate due to larger noise standard deviation.

## Conclusion

Computational systems biology increasingly relies on the heavy use of computational resources to improve the understanding of the complexity underlying cell biology. A widespread approach in computational systems biology is to specify a dynamical model of the biological process under investigation based on biochemical knowledge, and consider that the real system follows the same dynamics for some kinetic parameter values. Recent reports suggest that this has benefits in practical applications (*e.g.* [54]). Systematic implementations of the approach requires to deal with the fact that most kinetic parameters are often unknown, raising the issue of estimating these parameters from experimental data as efficiently as possible. An obvious sanity check is to recover kinetic parameters from synthetic data where dynamic and noise model are well specified, which is already quite a challenge.

In this paper we proposed a new general Bayesian OED strategy, and illustrated its relevance on an *in silico* biological network. The method takes advantage of the Bayesian framework to sequentially choose experiments to be performed, in order to estimate these parameters subject to cost constraints. The method relies on a single numerical criterion and does not depend on a specific instance of this problem. This is in our opinion a key point in order to reproducibly be able to deal with large scale networks of size comparable to of a cell for example. Experimental results suggest that the strategy has the potential to support experimental design in systems biology.

As noted by others [11,12,15-17], the approach focusing on kinetic parameter estimation is questionable. We also give empirical evidence that very different parameter values can produce very similar dynamical behaviors, potentially leading to non-identifiability issues. Moreover, focusing on parameter estimation supposes that the dynamical model represents the true underlying chemical process. In some cases, this might simply be false. For example, hypotheses underlying the law of mass action are not satisfied in the gene transcription process. However, simplified models might still be good proxies to characterize dynamical behaviors we are interested in. The real problem of interest is often to reproduce the dynamics of a system in terms of observable quantities, and to predict the system behavior for unseen manipulations. Parameters can be treated as latent variables which impact the dynamics of the system but cannot be observed. In this framework, the Bayesian formalism described here is well suited to tackle the problem of experimental design.

The natural continuity of this work is to adapt the method to treat larger problems. This raises computational issues and requires to develop numerical methods that scale well with the size of the problem. Sampling strategies that adapt to the local geometry and to multimodal aspects of the posterior, such as described *e.g.* in [55,56] are interesting directions to investigae in this context. The main bottlenecks are the cost of simulating large dynamical systems, and the need for large sample size in higher dimension for accurate posterior estimation. Posterior estimation in high dimensions is known to be hard and is an active subject of research. Although our Bayesian OED criterion is independent of the model investigated, it is likely that a good sampling strategy to implement may benefit from specific tuning in order to perform well on specific problem instances. As for reducing the computational burden of simulating large dynamical systems, promising research directions are parameter estimation methods that do not involve dynamical system simulation such as [57] or differential equation simulation methods that take into account both parameter uncertainty and numerical uncertainty such as the probabilistic integrator of [58].

## Availability of supporting data

An R package that allows to reproduce our results and simulations is available at the following URL: cran.r-project.org/package=pauwels2014.

## Additional files

Additional file 1
**Annex B.** Supplementary details regarding the sampling strategy used in our numerical experiments. The note also contains diagnosis information and marginal distribution samples to illustrate the efficacy of the sampling strategy in the setting of this paper.

Additional file 2
**Annex A.** PDF file. Description of the DREAM7 challenge network represented in Figure 2 and experimental design setting.
