# Supplementary material for: A Bayesian active learning strategy for sequential experimental design in systems biology
Source: BMC Syst Biol. 2014 Sep 26;8:102. doi: 10.1186/s12918-014-0102-6 (PMC4181721; doi:10.1186/s12918-014-0102-6)
Supplement: Additional file 1 — Annex B. Supplementary details regarding the sampling strategy used in our numerical experiments. The note also contains diagnosis information and marginal distribution samples to illustrate the efficacy of the sampling strategy in the setting of this paper. [file 12918_2014_102_MOESM1_ESM.pdf]

Annex B: Sampling strategy

This note describes the strategy used for parameter space exploration. We first describe the algorithmic details. We then show that this strategy provides efficient parameter exploration in our setting. In what follows, π_0 denotes a prior distribution, π denotes a posterior distribution and ω denotes the regularity enforcing term that is described in section 2.5.

1 A heuristic for parameter space exploration

The basic idea of the proposed strategy is to use multiple chains sampled using the Metropolis Hasting algorithm (MH). The transition kernel we used for each chain is an isotropic Gaussian with small variance combined with a (randomly chosen) single parameter Gaussian proposal with same variance. The variance of the proposal kernel was determined based on early simulations in order to obtain an acceptable trade-off between exploration and rejection rate. Despite the asymptotic consistency of the algorithm, in our setting of interest, practical implementation of this algorithm has the following shortcoming:

- Convergence to high density regions is slow;
- Single chains tend to get stuck in a single mode of the posterior distribution.

Both of these points constitute active subjects of research from theoretical and practical considerations. We propose here two simple heuristics to mitigate these flaws. They constitute a solution that proved to work well in our setting.

1.1 Use local optimization

In order to overcome the first point, we used local optimization to start each Markov chain at a high density point. The algorithm used is Broyden-Fletcher-Goldfarb-Shanno (BFGS) quasi-Newton method combined with finite difference approximation for first order information computation. The starting point is initialized by a draw from the prior π_0. During this phase, the regularity enforcing term ω is added to the log posterior in order to discard parameter regions corresponding to overly fast dynamics. The main reason for this choice is numerical stability during the optimization. The regularity enforcing term is only used at this point.

1.2 Consider multiple chains

In order to overcome the multimodality of the posterior surface, we considered repeating the procedure of the previous paragraph several times with random initialization (drawn from the prior π_0). The potential caveat of this approach lies in the recombination of the chains. Indeed, the initialization of each chain is the realization of a random process. Although each chain asymptotically converges to the true posterior, in practice, they remain in the posterior mode where they were initialized. Therefore, the process of recombining several chains should account for the initialization process. We barely have information about this process. Accounting for it would amount to compute the size of the bassin of attraction of each mode which is, in many aspects, harder than finding a mode. The heuristic we propose here is to approximate the

posterior by a mixture of Gaussian random variables, one for each mode, and to estimate the probability γ_i of falling in mode i accordingly.

Suppose that we have p samples, $S_1, \ldots, S_p$ drawn using the method described in the previous paragraph. Let μ_i denote the mean of S_i and Σ_i denote the covariance matrix of S_i for $i = 1, \ldots, p$. $\mathcal{N}(x, \mu, \Sigma)$ denotes the normal density with mean μ and variance Σ evaluated at point x. In order to estimate $\{\gamma_i\}_{i=1}^p$, we need to solve a linear system of the form

$$\sum_{j=1}^{p} \gamma_j \mathcal{N}(\mu_i, \mu_j, \Sigma_j) = \pi(\mu_i)\,, \quad i = 1 \ldots p\,,$$

where each γ_i is unknown. This equation arises by equating the density of a Gaussian mixture model (with unknown weights) with that of the posterior, when evaluated at the means, μ_i, of each sample S_i. There are p equations with p unknowns and the system is generically invertible. However, in practice, the posterior is only known up to a multiplicative factor. Moreover, it is possible that the solution of this equation does not lead to non-negative solutions, which, for our purpose, is limiting. We therefore consider the following estimation procedure. Define A to be the $p \times p$ matrix with $A_{ij} = \mathcal{N}(\mu_i, \mu_j, \Sigma_j)$, γ to be the p-vector of unknowns and Π the p-vector which entries are $\Pi_i = \pi(\mu_i)$. First solve the following optimization problem

$$\gamma^* = \min_{\gamma \geq 0} ||A\gamma - \Pi||^2, \tag{1}$$

where the inequality holds coordinate-wise. This can be solved *e.g.* using projected gradient descent. We then set

$$\gamma_i = \frac{\gamma_i^*}{\sum_j \gamma_j^*}. \tag{2}$$

Figure S1 shows that the procedure sucessfully identifies mixing weights proportions when the Gaussian mixture model assumption is well specified. The number of modes p specifies a tradeoff between computational cost and exploration accuracy.

1.3 Final algorithm

Combining the two procedures described in the previous sections results in Algorithm 1. This details the `sample` function used in the main text.

Algorithm 1: sample

input : Posterior π (evaluation up to a multiplicative factor), prior π_0, regularity enforcing term ω, number of subsamples p

output: $\{\theta_i\}_{i=1}^N$

```
begin
    for i = 1 to p do
        θ_temp ~ π_0
        θ_0^i ← BFGS(θ_temp, ω)
        S_i ← MH(θ_0^i)
    Estimate {γ_i}_{i=1}^p using (2)
    {θ_i}_{i=1}^N ← sub-sample({S_i}_{i=1}^p, {γ_i}_{i=1}^p)
```

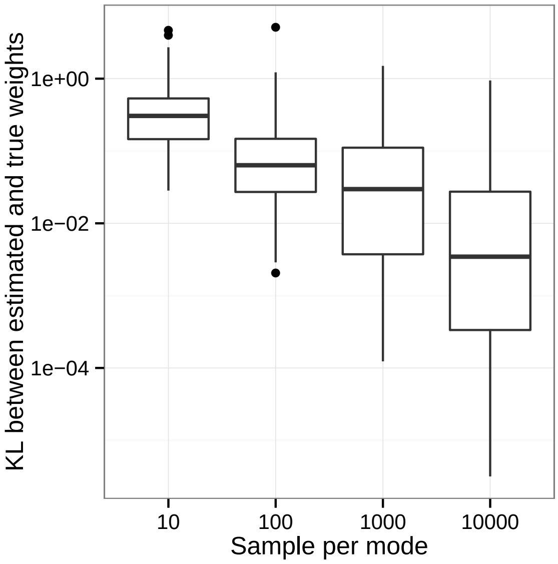

Figure S1: Simulation regarding the weight estimation procedure in dimension 4 with a mixture of 6 Gaussians. For each experiment, means, variances and mixing weights are drawn randomly from unit Gaussian, unit Wishart and unit Dirichlet (uniform on the simplex) distributions. We draw a sample from each mode, estimate the weights using (2) and compare them with the weights that generated the mixture distribution. We vary the sample size in each mode and measure the KL divergence between estimated and true mixing weights proportions (for multinomial distributions with parameters $\alpha, \beta \in \mathbb{R}^p$ we have $KL(\alpha, \beta) = \sum_{i=1}^{p} \alpha_i \log\left(\frac{\alpha_i}{\beta_i}\right)$). The boxplots represent 100 simulation for each sample size.

2 Diagnostic and results

In order to verify that the method described in the previous section allows efficient parameter space exploration, we ran it ten times on the same posterior distribution with different initial random seeds. This results in ten samples: $samp_1 \dots samp_0$ drawn using Algorithm 1. For each parameter, we compare the dispersion of each sample compared to the dispersion of the concatenation of all ten samples, when we fix the number of modes to 20. The results are presented in Figure S2 which shows, for each parameter, the ratio between the standard deviation carried by each sample and the total standard deviation, i.e., the standard deviation of the concatenation of all samples. When this quantity is close to 1, this means that both mean and dispersion are comparable between different samples. The plot suggests that the proposed parameter space exploration strategy succeeds in reproducibly exploring the parameter space. The corresponding marginal densities are represented in Figure S3. It should be noticed that the posterior marginals are coherent between different runs.

2.1 A word on identifiability

In our framework, lack of identifiability manifests itself though flat likelihood surfaces. Since we adopt a Bayesian point of view, the result is that the marginal distributions related to non-identifiable quantities are close to their prior distribution. This is seen in Figure S3 where at the beginning of the process, most parameters are not identifiable. This results in spread posterior samples on a wide range of values. In this context, identifiability generates dispertions between prior and posterior distributions through a reduction of the dispersion.

To the classical notion of identifiable versus non-identifiable parameters, Bayesian analysis

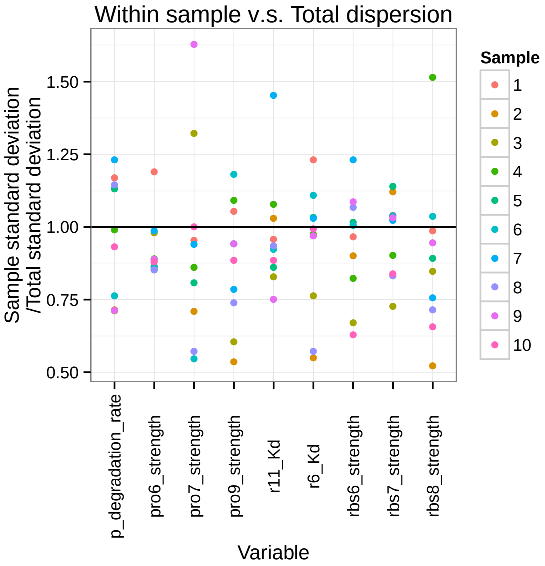

Figure S2: Ratio between the standard deviation carried by each sample and the total standard deviation, for each parameter.

substitutes a continum of identifiability statuses reflected by the dispersion of their posterior distributions. It is of interest to notice that we did not explicitely ask for parameter identifiability statuses. In particular, we illustrate it on single parameter values because they are physical quantities for which identifiability analysis is usually carried out. Similar effects may be detected through Bayesian analysis for quatities that are not defined *à priori*, such as linear (or non linear) combination of parameters in our context.

Moreover, Figure S3 shows that posterior distributions, after the collection of data through the design proccess, are very concentrated. This illustrates how the design process affects the uncertainty in parameter values. Figure S4 shows details of posterior marginal during the design process for the first simulation (first column of Figure S3). This illustrate the fact that the experimental design method allows to alleviate non-identifiabilities by considering informative experiments. Indeed, this results in posterior concentration, and divergence from the prior, as more experiments are carried out.

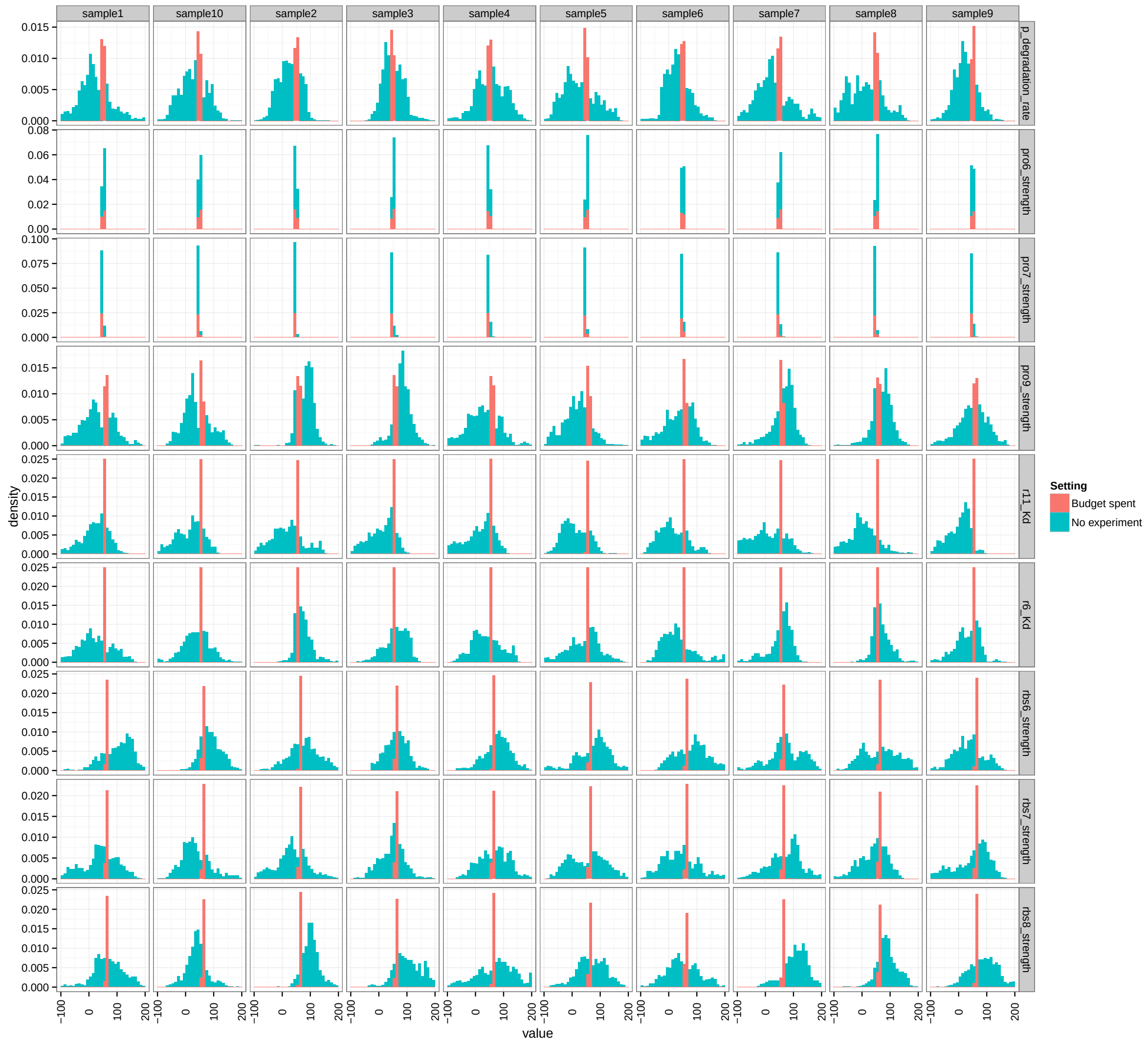

Figure S3: Marginal distributions for each sample, and each parameter, before (blue) and after (red) experimental design. The red density is divided by 4 for visual display. These are the same samples based on which quantities displayed in figure S2 where computed. For most parameters, the dispersion correspond to the standard error of the prior (100). Note that the parameter values are displayed on log scale. This corresponds to physical values ranging from 10^{-7} to 10^{5} for the first parameter and from 10^{-9} to 10^{9} for the other ones.

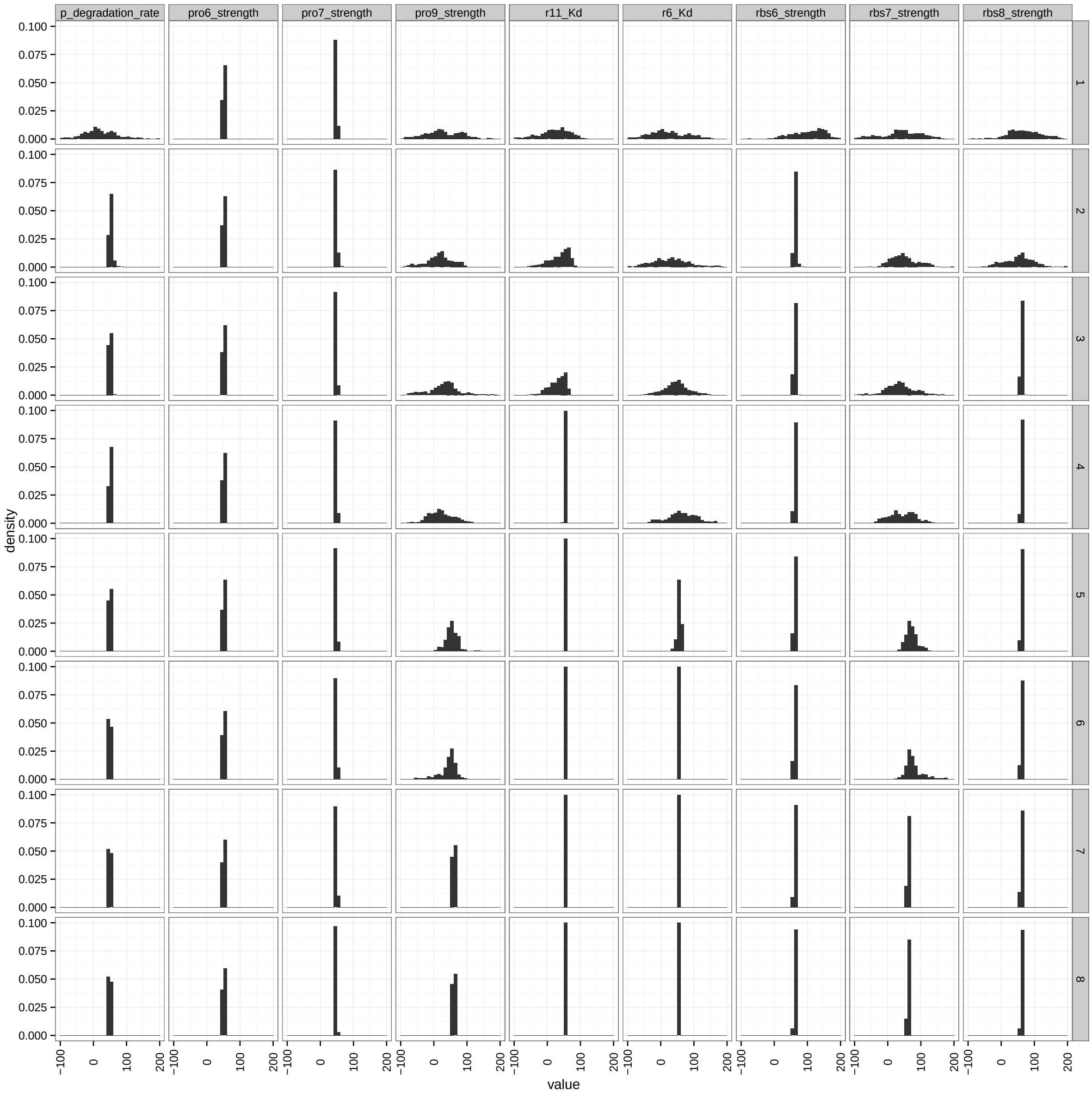

Figure S4: Evolution of marginals for one experimental design simulation (first column in figure S3). Each line correspond to the collection of data from one experiment. The figure illustrate the mechanism underlying the Bayesian design procedure, as non identifiabilities are alleviated (by choice of experiments), the posterior distributions concentrate around a mode.
