# Supplementary material for: A Bayesian active learning strategy for sequential experimental design in systems biology
Source: BMC Syst Biol. 2014 Sep 26;8:102. doi: 10.1186/s12918-014-0102-6 (PMC4181721; doi:10.1186/s12918-014-0102-6)
Supplement: Additional file 2 — Annex A. PDF file. Description of the DREAM7 challenge network represented in Figure 2 and experimental design setting. [file 12918_2014_102_MOESM2_ESM.pdf]

Annex A: DREAM7 network description

1 Kinetics

The complete network description and implementations of integrators to simulate its dynamics are available from DREAM7 challenge website. The dynamical equations governing the evolution of time concentration are as follows, where the names of the different variables are represented in Figure 2.

$$[\mathrm{as1}] = \frac{\left(\frac{[\mathrm{p1}]}{\mathrm{r1}_{\mathrm{Kd}}}\right)^{\mathrm{r1}_{\mathrm{h}}}}{1+\left(\frac{[\mathrm{p1}]}{\mathrm{r1}_{\mathrm{Kd}}}\right)^{\mathrm{r1}_{\mathrm{h}}}}$$

$$[\mathrm{as2}] = \frac{\left(\frac{[\mathrm{p1}]}{\mathrm{r2}_{\mathrm{Kd}}}\right)^{\mathrm{r2}_{\mathrm{h}}}}{1+\left(\frac{[\mathrm{p1}]}{\mathrm{r2}_{\mathrm{Kd}}}\right)^{\mathrm{r2}_{\mathrm{h}}}}$$

$$[\mathrm{as3}] = \frac{\left(\frac{[\mathrm{p4}]}{\mathrm{r5}_{\mathrm{Kd}}}\right)^{\mathrm{r5}_{\mathrm{h}}}}{1+\left(\frac{[\mathrm{p4}]}{\mathrm{r5}_{\mathrm{Kd}}}\right)^{\mathrm{r5}_{\mathrm{h}}}}$$

$$[\mathrm{as5}] = \frac{\left(\frac{[\mathrm{p8}]}{\mathrm{r13}_{\mathrm{Kd}}}\right)^{\mathrm{r13}_{\mathrm{h}}}}{1+\left(\frac{[\mathrm{p8}]}{\mathrm{r13}_{\mathrm{Kd}}}\right)^{\mathrm{r13}_{\mathrm{h}}}}$$

$$[\mathrm{as6}] = \frac{\left(\frac{[\mathrm{p9}]}{\mathrm{r9}_{\mathrm{Kd}}}\right)^{\mathrm{r9}_{\mathrm{h}}}}{1+\left(\frac{[\mathrm{p9}]}{\mathrm{r9}_{\mathrm{Kd}}}\right)^{\mathrm{r9}_{\mathrm{h}}}}$$

$$[\mathrm{as7}] = \frac{\left(\frac{[\mathrm{p6}]}{\mathrm{r12}_{\mathrm{Kd}}}\right)^{\mathrm{r12}_{\mathrm{h}}}}{1+\left(\frac{[\mathrm{p6}]}{\mathrm{r12}_{\mathrm{Kd}}}\right)^{\mathrm{r12}_{\mathrm{h}}}}$$

$$[\mathrm{as9}] = \frac{\left(\frac{[\mathrm{p6}]}{\mathrm{r11}_{\mathrm{Kd}}}\right)^{\mathrm{r11}_{\mathrm{h}}}}{1+\left(\frac{[\mathrm{p6}]}{\mathrm{r11}_{\mathrm{Kd}}}\right)^{\mathrm{r11}_{\mathrm{h}}}}$$

$$[\mathrm{rs1a}] = \frac{1}{1+\left(\frac{[\mathrm{p2}]}{\mathrm{r4}_{\mathrm{Kd}}}\right)^{\mathrm{r4}_{\mathrm{h}}}}$$

$$[\mathrm{rs1b}] = \frac{1}{1+\left(\frac{[\mathrm{p6}]}{\mathrm{r8}_{\mathrm{Kd}}}\right)^{\mathrm{r8}_{\mathrm{h}}}}$$

$$[\mathrm{rs2}] = \frac{1}{1+\left(\frac{[\mathrm{p3}]}{\mathrm{r3}_{\mathrm{Kd}}}\right)^{\mathrm{r3}_{\mathrm{h}}}}$$

$$[\mathrm{rs3}] = \frac{1}{1+\left(\frac{[\mathrm{p5}]}{\mathrm{r7}_{\mathrm{Kd}}}\right)^{\mathrm{r7}_{\mathrm{h}}}}$$

$$[\mathrm{rs7}] = \frac{1}{1+\left(\frac{[\mathrm{p7}]}{\mathrm{r6}_{\mathrm{Kd}}}\right)^{\mathrm{r6}_{\mathrm{h}}}}$$

$$[\mathrm{rs8}] = \frac{1}{1+\left(\frac{[\mathrm{p9}]}{\mathrm{r10}_{\mathrm{Kd}}}\right)^{\mathrm{r10}_{\mathrm{h}}}}$$

$$\begin{aligned}
[\mathrm{g1}] &= [\mathrm{as1}] \cdot [\mathrm{rs1a}] \cdot [\mathrm{rs1b}] \\
[\mathrm{g2}] &= [\mathrm{as2}] \cdot [\mathrm{rs2}] \\
[\mathrm{g3}] &= [\mathrm{as3}] \cdot [\mathrm{rs3}] \\
[\mathrm{g4}] &= [\mathrm{as4}] \cdot [\mathrm{rs4}] \\
[\mathrm{g5}] &= [\mathrm{as5}] \\
[\mathrm{g6}] &= 1 \\
[\mathrm{g7}] &= [\mathrm{as7}] \cdot [\mathrm{rs7}] \\
[\mathrm{g8}] &= [\mathrm{rs8}] \\
[\mathrm{g9}] &= [\mathrm{as9}]
\end{aligned}$$

$$\frac{\mathrm{d}\left([\mathrm{p1}]\right)}{\mathrm{d}t} = \mathrm{rbs1}_{\mathrm{strength}} \cdot [\mathrm{v1}_{\mathrm{mrna}}] - \mathrm{p1}_{\mathrm{degradationRate}} \cdot [\mathrm{p1}]$$

$$\frac{\mathrm{d}\left([\mathrm{p1}]\right)}{\mathrm{d}t} = \mathrm{rbs1}_{\mathrm{strength}} \cdot [\mathrm{v1}_{\mathrm{mrna}}] - \mathrm{p1}_{\mathrm{degradationRate}} \cdot [\mathrm{p1}]$$

$$\frac{\mathrm{d}\left([\mathrm{p2}]\right)}{\mathrm{d}t} = \mathrm{rbs2}_{\mathrm{strength}} \cdot [\mathrm{v2}_{\mathrm{mrna}}] - \mathrm{p2}_{\mathrm{degradationRate}} \cdot [\mathrm{p2}]$$
$$\frac{\mathrm{d}\left([\mathrm{p3}]\right)}{\mathrm{d}t} = \mathrm{rbs3}_{\mathrm{strength}} \cdot [\mathrm{v3}_{\mathrm{mrna}}] - \mathrm{p3}_{\mathrm{degradationRate}} \cdot [\mathrm{p3}]$$
$$\frac{\mathrm{d}\left([\mathrm{p4}]\right)}{\mathrm{d}t} = \mathrm{rbs4}_{\mathrm{strength}} \cdot [\mathrm{v4}_{\mathrm{mrna}}] - \mathrm{p4}_{\mathrm{degradationRate}} \cdot [\mathrm{p4}]$$
$$\frac{\mathrm{d}\left([\mathrm{p5}]\right)}{\mathrm{d}t} = \mathrm{rbs5}_{\mathrm{strength}} \cdot [\mathrm{v5}_{\mathrm{mrna}}] - \mathrm{p5}_{\mathrm{degradationRate}} \cdot [\mathrm{p5}]$$
$$\frac{\mathrm{d}\left([\mathrm{p6}]\right)}{\mathrm{d}t} = \mathrm{rbs6}_{\mathrm{strength}} \cdot [\mathrm{v6}_{\mathrm{mrna}}] - \mathrm{p6}_{\mathrm{degradationRate}} \cdot [\mathrm{p6}]$$
$$\frac{\mathrm{d}\left([\mathrm{p7}]\right)}{\mathrm{d}t} = \mathrm{rbs8}_{\mathrm{strength}} \cdot [\mathrm{v7}_{\mathrm{mrna}}] - \mathrm{p7}_{\mathrm{degradationRate}} \cdot [\mathrm{p7}]$$
$$\frac{\mathrm{d}\left([\mathrm{p8}]\right)}{\mathrm{d}t} = \mathrm{rbs7}_{\mathrm{strength}} \cdot [\mathrm{v8}_{\mathrm{mrna}}] - \mathrm{p8}_{\mathrm{degradationRate}} \cdot [\mathrm{p8}]$$
$$\frac{\mathrm{d}\left([\mathrm{p9}]\right)}{\mathrm{d}t} = \mathrm{rbs9}_{\mathrm{strength}} \cdot [\mathrm{v9}_{\mathrm{mrna}}] - \mathrm{p9}_{\mathrm{degradationRate}} \cdot [\mathrm{p9}]$$
$$\frac{\mathrm{d}\left([\mathrm{v1}_{\mathrm{mrna}}]\right)}{\mathrm{d}t} = \mathrm{pro1}_{\mathrm{strength}} \cdot [\mathrm{g1}] - \mathrm{v1}_{\mathrm{mrnaDegradationRate}} \cdot [\mathrm{v1}_{\mathrm{mrna}}]$$
$$\frac{\mathrm{d}\left([\mathrm{v2}_{\mathrm{mrna}}]\right)}{\mathrm{d}t} = \mathrm{pro2}_{\mathrm{strength}} \cdot [\mathrm{g2}] - \mathrm{v2}_{\mathrm{mrnaDegradationRate}} \cdot [\mathrm{v2}_{\mathrm{mrna}}]$$
$$\frac{\mathrm{d}\left([\mathrm{v3}_{\mathrm{mrna}}]\right)}{\mathrm{d}t} = \mathrm{pro3}_{\mathrm{strength}} \cdot [\mathrm{g3}] - \mathrm{v3}_{\mathrm{mrnaDegradationRate}} \cdot [\mathrm{v3}_{\mathrm{mrna}}]$$
$$\frac{\mathrm{d}\left([\mathrm{v4}_{\mathrm{mrna}}]\right)}{\mathrm{d}t} = \mathrm{pro4}_{\mathrm{strength}} \cdot [\mathrm{g4}] - \mathrm{v4}_{\mathrm{mrnaDegradationRate}} \cdot [\mathrm{v4}_{\mathrm{mrna}}]$$
$$\frac{\mathrm{d}\left([\mathrm{v5}_{\mathrm{mrna}}]\right)}{\mathrm{d}t} = \mathrm{pro5}_{\mathrm{strength}} \cdot [\mathrm{g5}] - \mathrm{v5}_{\mathrm{mrnaDegradationRate}} \cdot [\mathrm{v5}_{\mathrm{mrna}}]$$
$$\frac{\mathrm{d}\left([\mathrm{v6}_{\mathrm{mrna}}]\right)}{\mathrm{d}t} = \mathrm{pro6}_{\mathrm{strength}} \cdot [\mathrm{g6}] - \mathrm{v6}_{\mathrm{mrnaDegradationRate}} \cdot [\mathrm{v6}_{\mathrm{mrna}}]$$
$$\frac{\mathrm{d}\left([\mathrm{v7}_{\mathrm{mrna}}]\right)}{\mathrm{d}t} = \mathrm{pro7}_{\mathrm{strength}} \cdot [\mathrm{g9}] - \mathrm{v7}_{\mathrm{mrnaDegradationRate}} \cdot [\mathrm{v7}_{\mathrm{mrna}}]$$
$$\frac{\mathrm{d}\left([\mathrm{v8}_{\mathrm{mrna}}]\right)}{\mathrm{d}t} = \mathrm{pro9}_{\mathrm{strength}} \cdot [\mathrm{g7}] - \mathrm{v8}_{\mathrm{mrnaDegradationRate}} \cdot [\mathrm{v8}_{\mathrm{mrna}}]$$
$$\frac{\mathrm{d}\left([\mathrm{v9}_{\mathrm{mrna}}]\right)}{\mathrm{d}t} = \mathrm{pro8}_{\mathrm{strength}} \cdot [\mathrm{g8}] - \mathrm{v9}_{\mathrm{mrnaDegradationRate}} \cdot [\mathrm{v9}_{\mathrm{mrna}}]$$

With kinetic variables

$[\mathrm{p1}], [\mathrm{p2}], [\mathrm{p3}], [\mathrm{p4}], [\mathrm{p5}], [\mathrm{p6}], [\mathrm{p7}], [\mathrm{p8}], [\mathrm{p9}]$

$[\mathrm{v1}_{\mathrm{mrna}}], [\mathrm{v2}_{\mathrm{mrna}}], [\mathrm{v3}_{\mathrm{mrna}}], [\mathrm{v4}_{\mathrm{mrna}}], [\mathrm{v5}_{\mathrm{mrna}}], [\mathrm{v6}_{\mathrm{mrna}}], [\mathrm{v7}_{\mathrm{mrna}}], [\mathrm{v8}_{\mathrm{mrna}}], [\mathrm{v9}_{\mathrm{mrna}}],$

And kinetic parameters

$\mathrm{v1}_{\mathrm{mrnaDegradationRate}}$
$\mathrm{v2}_{\mathrm{mrnaDegradationRate}}$
$\mathrm{v7}_{\mathrm{mrnaDegradationRate}}$
$\mathrm{v6}_{\mathrm{mrnaDegradationRate}}$
$\mathrm{v5}_{\mathrm{mrnaDegradationRate}}$
$\mathrm{v6}_{\mathrm{mrnaDegradationRate}}$
$\mathrm{v7}_{\mathrm{mrnaDegradationRate}}$
$\mathrm{v8}_{\mathrm{mrnaDegradationRate}}$
$\mathrm{v9}_{\mathrm{mrnaDegradationRate}}$
$\mathrm{p1}_{\mathrm{degradationRate}}$
$\mathrm{p2}_{\mathrm{degradationRate}}$
$\mathrm{p3}_{\mathrm{degradationRate}}$
$\mathrm{p4}_{\mathrm{degradationRate}}$
$\mathrm{p5}_{\mathrm{degradationRate}}$
$\mathrm{p6}_{\mathrm{degradationRate}}$
$\mathrm{p7}_{\mathrm{degradationRate}}$
$\mathrm{p9}_{\mathrm{degradationRate}}$
$\mathrm{p8}_{\mathrm{degradationRate}}$
$\mathrm{pro1}_{\mathrm{strength}}$
$\mathrm{pro2}_{\mathrm{strength}}$
$\mathrm{pro3}_{\mathrm{strength}}$
$\mathrm{pro4}_{\mathrm{strength}}$
$\mathrm{pro5}_{\mathrm{strength}}$
$\mathrm{pro6}_{\mathrm{strength}}$
$\mathrm{pro7}_{\mathrm{strength}}$
$\mathrm{pro8}_{\mathrm{strength}}$
$\mathrm{pro9}_{\mathrm{strength}}$
$\mathrm{rbs1}_{\mathrm{strength}}$
$\mathrm{rbs2}_{\mathrm{strength}}$
$\mathrm{rbs3}_{\mathrm{strength}}$
$\mathrm{rbs4}_{\mathrm{strength}}$
$\mathrm{rbs5}_{\mathrm{strength}}$
$\mathrm{rbs6}_{\mathrm{strength}}$
$\mathrm{rbs7}_{\mathrm{strength}}$
$\mathrm{rbs8}_{\mathrm{strength}}$
$\mathrm{rbs9}_{\mathrm{strength}}$
$\mathrm{r1}_{\mathrm{Kd}}, \mathrm{r1}_{\mathrm{h}}$
$\mathrm{r2}_{\mathrm{Kd}}, \mathrm{r2}_{\mathrm{h}}$
$\mathrm{r3}_{\mathrm{Kd}}, \mathrm{r3}_{\mathrm{h}}$
$\mathrm{r4}_{\mathrm{Kd}}, \mathrm{r4}_{\mathrm{h}}$
$\mathrm{r5}_{\mathrm{Kd}}, \mathrm{r5}_{\mathrm{h}}$
$\mathrm{r6}_{\mathrm{Kd}}, \mathrm{r6}_{\mathrm{h}}$
$\mathrm{r7}_{\mathrm{Kd}}, \mathrm{r6}_{\mathrm{h}}$
$\mathrm{r8}_{\mathrm{Kd}}, \mathrm{r7}_{\mathrm{h}}$
$\mathrm{r9}_{\mathrm{Kd}}, \mathrm{r9}_{\mathrm{h}}$
$\mathrm{r10}_{\mathrm{Kd}}, \mathrm{r10}_{\mathrm{h}}$
$\mathrm{r11}_{\mathrm{Kd}}, \mathrm{r11}_{\mathrm{h}}$
$\mathrm{r12}_{\mathrm{Kd}}, \mathrm{r12}_{\mathrm{h}}$
$\mathrm{r13}_{\mathrm{Kd}}, \mathrm{r13}_{\mathrm{h}}$

Among them, we suppose that we have

$$\begin{aligned} & \mathrm{v1}_{\mathrm{mrnaDegradationRate}} = \mathrm{v2}_{\mathrm{mrnaDegradationRate}} = \mathrm{v3}_{\mathrm{mrnaDegradationRate}} \\ =& \mathrm{v4}_{\mathrm{mrnaDegradationRate}} = \mathrm{v5}_{\mathrm{mrnaDegradationRate}} = \mathrm{v6}_{\mathrm{mrnaDegradationRate}} \\ =& \mathrm{v7}_{\mathrm{mrnaDegradationRate}} = \mathrm{v8}_{\mathrm{mrnaDegradationRate}} = \mathrm{v9}_{\mathrm{mrnaDegradationRate}} \\ =& 1, \end{aligned}$$

and

$$\begin{aligned} & \mathrm{p1}_{\mathrm{degradationRate}} = \mathrm{p2}_{\mathrm{degradationRate}} = \mathrm{p3}_{\mathrm{degradationRate}} \\ =& \mathrm{p4}_{\mathrm{degradationRate}} = \mathrm{p5}_{\mathrm{degradationRate}} = \mathrm{p6}_{\mathrm{degradationRate}} \\ =& \mathrm{p7}_{\mathrm{degradationRate}} = \mathrm{p8}_{\mathrm{degradationRate}} = \mathrm{p9}_{\mathrm{degradationRate}}. \end{aligned}$$

This makes a total of 45 parameters governing the behaviour of 18 kinetic variables.

2 Description of the experimental cost and challege experimental plan

Perturbation	Cost
Wildtype<br>(no perturbation)	0
Gene knockdown<br>(mrna degradation rate $\times 10$)	350
Decrease ribosomal activity<br>(ribosomal strength /10)	450
Delete gene<br>(mrna and protein concentration = 0)	800

Concentration to observe	Cost
Two proteins<br>(Resolution: $0.5s$)	300
All mrna<br>(Resolution: $4s$)	500
All mrna<br>(Resolution: $2s$)	1000

Table S1: Description of the cost. Each participant begins with a 10000 initial credit budget. The cost of each experiment was the sum of the costs of the corresponding molecular perturbation and quantity to observe. Molecular perturbations could be performed on any of the genes.

Perturbation	Observation
Wildtype	mrna low resolution (free)
Wildtype	pairwise protein time course for all proteins
Delete gene 7	Protein 3 and 8
Decrease ribosomal strength gene 9	All mrna at high resolution
Knockdown gene 7	Protein 8 and 9
Knockdown gene 6	Protein 3 and 8
Knockdown gene 4	Protein 3 and 7
Decrease ribowomal strength gene 6	All mrna at high resolution
Delete gene 9	Protein 2 and 3

Table S2: Successive experiments performed during the challenge.

Perturbation	Observation
Divide $r9_{Kd}$ by 10<br>Multiply $rbs3_{strength}$ by 2<br>Multiply $rbs3_{strength}$ by 10	Proteins 3, 5 and 8<br>$0.5s$ time resolution<br>from $t = 0$ to $t = 20$

Table S3: Unseen experiment.
